# Management of Extensive Buccosinusal and Bucconasal Communications After Maxillary Giant Cell Tumor Resection Using the Cortical Bone Lamina Technique: A Case Report

**DOI:** 10.7759/cureus.59180

**Published:** 2024-04-27

**Authors:** Selin Güney, Nicolas Jaballah, Clotaire Kati Coulibaly

**Affiliations:** 1 Department of Stomatology and Maxillofacial Surgery, Novo Hospital, Pontoise, FRA

**Keywords:** bone lamina technique, oral defect reconstruction, bucco-nasal communication, bucco-sinusal communication, oral histopathology, giant-cell tumor

## Abstract

Giant cell tumors are rare, locally aggressive non-odontogenic osteolytic tumors associated with high rates of local recurrence. Treatment modalities are subject to considerable controversy, with successful outcomes hinging on achieving complete tumor elimination through thorough curettage. A 78-year-old male referred in December 2023 for a persistent mucosal lesion in the right maxilla under a removable denture. Clinical examination revealed a well-defined erythematous nodular lesion measuring approximately 3 cm along its long axis, localized on ridge quadrant 1. Biopsy confirmed the diagnosis of giant cell tumor.

Although complete resection with healthy margins may be justified for aggressive lesions, it often results in significant morbidity and requires immediate defect reconstruction. Some studies suggest favorable long-term outcomes with guided bone regeneration (GBR). The bone lamina technique uses a xenogeneic cortical bone membrane to maintain space and promote bone healing. This surgical approach promotes bone healing through the mechanical support and biological properties of the lamina.

The purpose of this case report is to evaluate the efficacy of the bone lamina technique and its role in managing complications following giant cell tumor resection.

## Introduction

Giant cell tumors (GCTs) are rare, locally aggressive, and progressive osteolytic lesions for which no medical or surgical reference exists [1]. Highly recurrent, one of the treatment strategies usually consists of intralesional injection of corticoids to limit the lesion’s size and reduce the need for extensive surgical resection, which may result in severe aesthetic and functional defects [2,3]. However, this frequently results in large and extensive resection due to the aggressiveness of the lesion. Post-surgical reconstruction of the bone defect must be considered to limit postoperative sequelae.

Various clinical parameters, such as the size, shape, and dimensions of the defect, determine the choice of surgical treatment. Some studies have reported favorable long-term outcomes with guided bone regeneration (GBR) [4]. The use of autogenous bone substitutes for defect reconstruction has shown promising clinical results. However, donor site morbidity, the limited quantity of graft available, and unpredictable resorption are leading us to consider more appropriate strategies [5].

More recently, the bone lamina technique for defect reconstruction was described by Wachtel et al. In their study, they utilized a xenogeneic cortical bone shield in combination with particulate bone substitute and a thin collagen barrier to maintain space and promote bone healing. This surgical approach resulted in bone healing through the mechanical and biological properties of the lamina [6].

Therefore, the purpose of this case report is to assess the closure of buccosinusal and bucconasal communications and monitor the bone healing process after giant cell tumor resection using the cortical bone lamina technique.

## Case presentation

**Figure 1:**
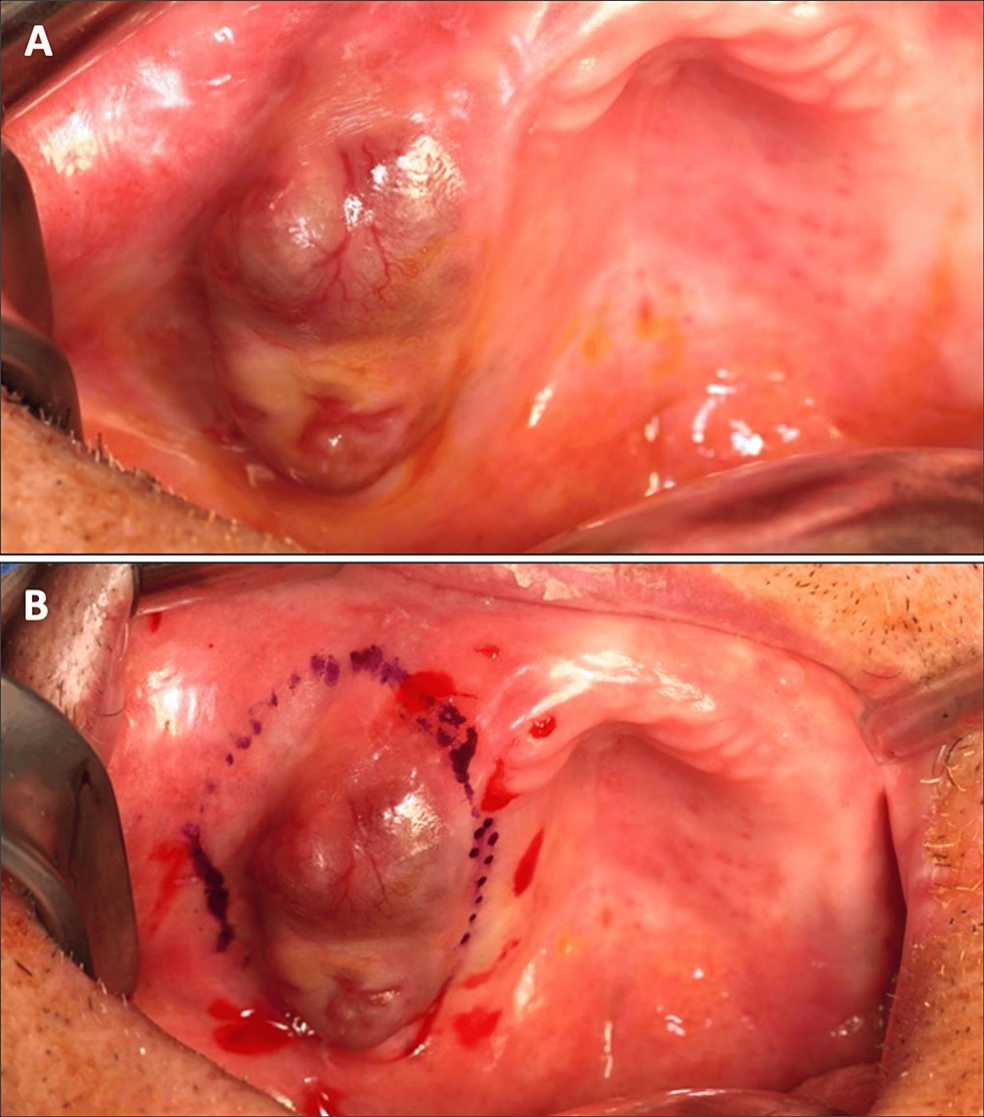
Clinical situation (A) The nodular lesion could be observed on the ridge (B) Delimitation of surgical margins.

**Figure 2:**
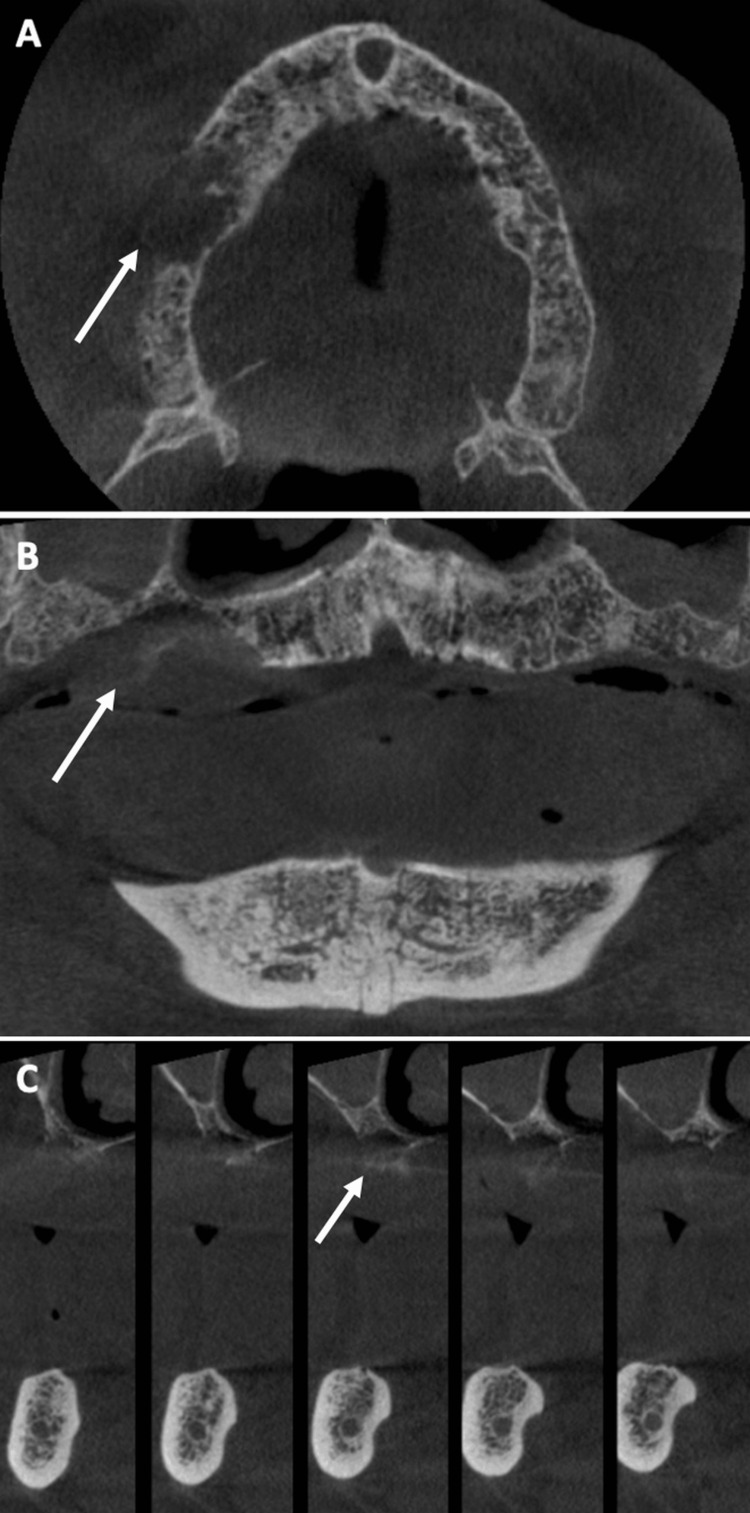
Large osteolysis area could be observed in (A) axial view, (B) frontal view and (C) tangential views of CT scan.

A 78-year-old male patient (ASA III) was referred by his dentist in December 2023 for a persistent mucosal lesion localized in the right maxilla under a removable denture. The patient has a history of hypertension and chronic obstructive pulmonary disease (COPD). His main medications were a combination of losartan and hydrochlorothiazide, formoterol fumarate, tiotropium, pravastatin, and acetylsalicylic acid. The chief complaint is instability and pain associated with wearing a removable denture that has been in place for six weeks. Clinical examination showed a well-delimited nodular lesion with firm consistency, without bleeding on contact, measuring approximately 3 cm along its long axis, located on the ridge quadrant 1 (Figure 1A, 1B). No cervical lymphadenopathy was detected. Computed Tomography (CT) scan revealed a large osteolytic area on the right alveolar ridge in the axial (Figure 2A), frontal (Figure 2B), and tangential views (Figure 2C). The rest of the oral mucosa or oral structures were normal. Clinical symptoms such as pain and swelling, combined with radiological findings indicating the osteolytic and expansive nature of the bone lesion, resulted in various preliminary diagnoses, including epidermoid carcinoma, lymphoma, and central giant cell granuloma. However, the etiological diagnosis could only be confirmed through histopathological analysis.

The treatment was carried out in four steps: (I) Biopsy for anatomopathological analysis to confirm the diagnostic hypothesis. (II) Resection of mucosal and bone lesion with thorough and complete curettage to healthy margins. (III) Closure of communications and reconstruction of bone defects using xenogeneic lamina membranes fixed by osteosynthesis screws. (IV) Post-operative prescriptions and scheduled clinical and radiographic follow-up.

**Figure 3:**
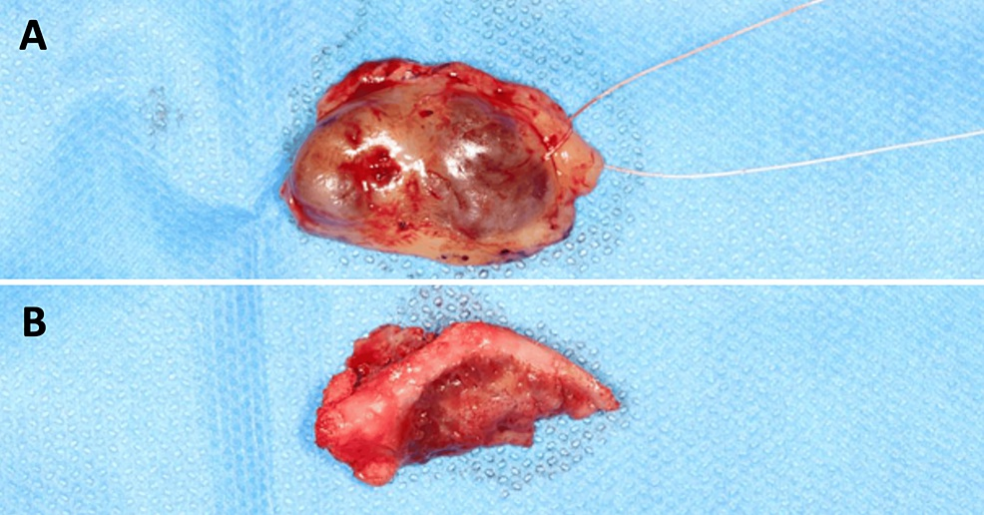
Surgical pieces for (A) mucosal excision and (B) bone resection.

**Figure 4:**
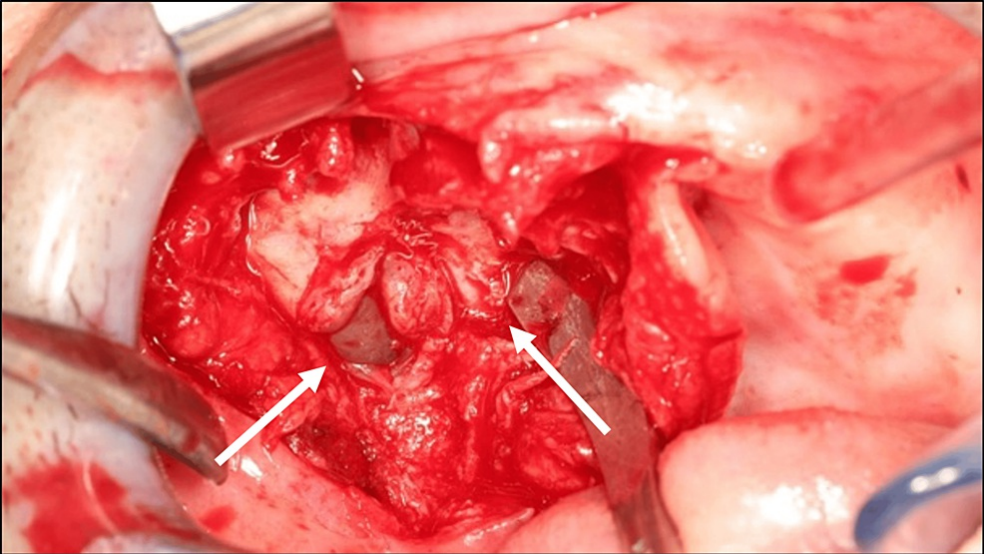
Surgical site after resection and bone curettage. Buccosinusal and bucconasal communications could be observed.

Before treatment, free and informed consent was obtained from the patient. A biopsy was performed and confirmed the diagnosis of a GCT. The lesion was resected to healthy margins under general anesthesia, using a #15 scalpel blade for the mucosa and a piezotome for the bone extension of the lesion (Figure 3). After bone resection, buccosinusal and bucconasal communications were identified intraoperatively due to the size and extent of the resection (Figure 4).

**Figure 5:**
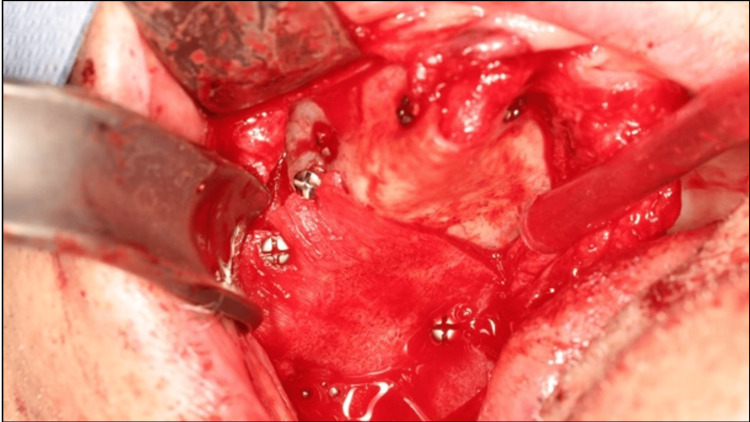
Application of lamina membranes and fixation with osteosynthesis screws.

**Figure 6:**
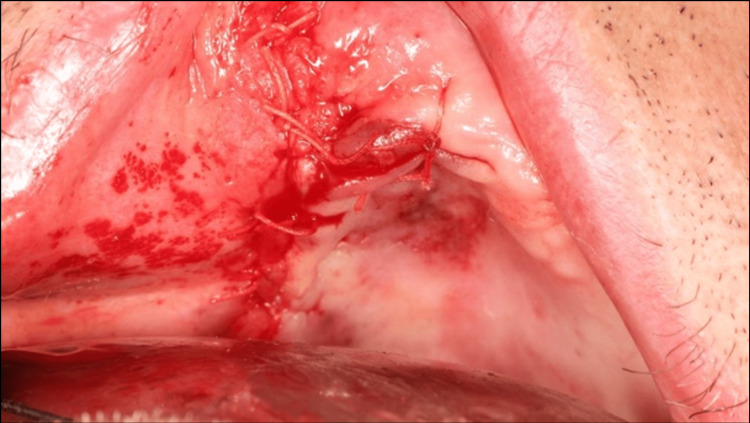
Primary wound closure of surgical site.

Buccosinusal and bucconasal communications were closed using two xenogeneic lamina membranes fixed with six osteosynthesis screws (Figure 5). A periosteal releasing incision provided sufficient tissue laxity to close the wound by primary intention, thus preventing secondary exposure of the biomaterial or osteosynthesis screws (Figure 6).

**Figure 7:**
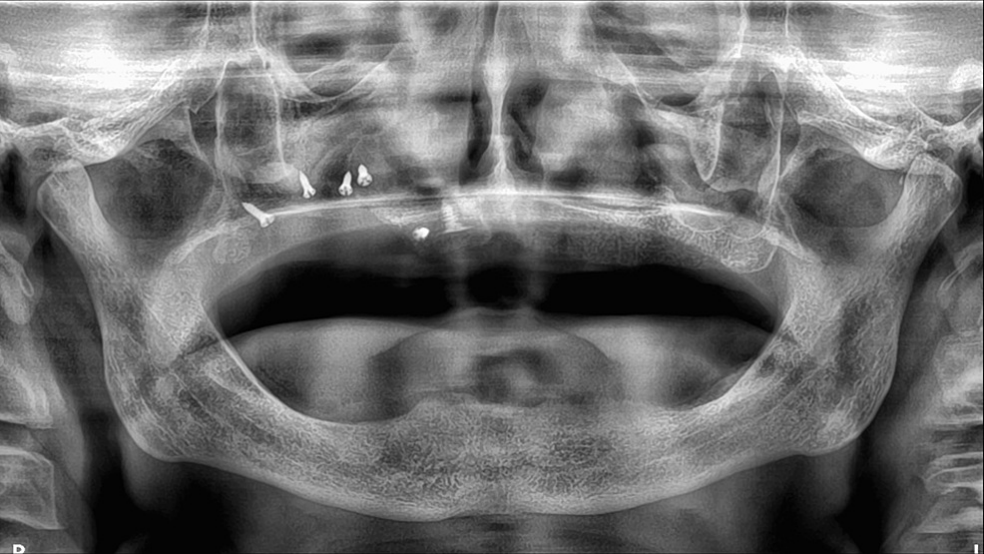
Immediate post-operative panoramic X-ray.

Post-operative prescriptions of amoxicillin-clavulanic acid, paracetamol-codeine combination, and chlorhexidine solution were provided. Post-operative checks at seven, 15, and 30 days were performed. Surveillance of the surgical site was essential to ensure that the lamina bone barrier was not exposed and to remind the patient not to wear the removable denture during wound and bone healing. An immediate post-operative panoramic X-ray was taken (Figure 7). A CT scan check-up is also scheduled at four months.

**Figure 8:**
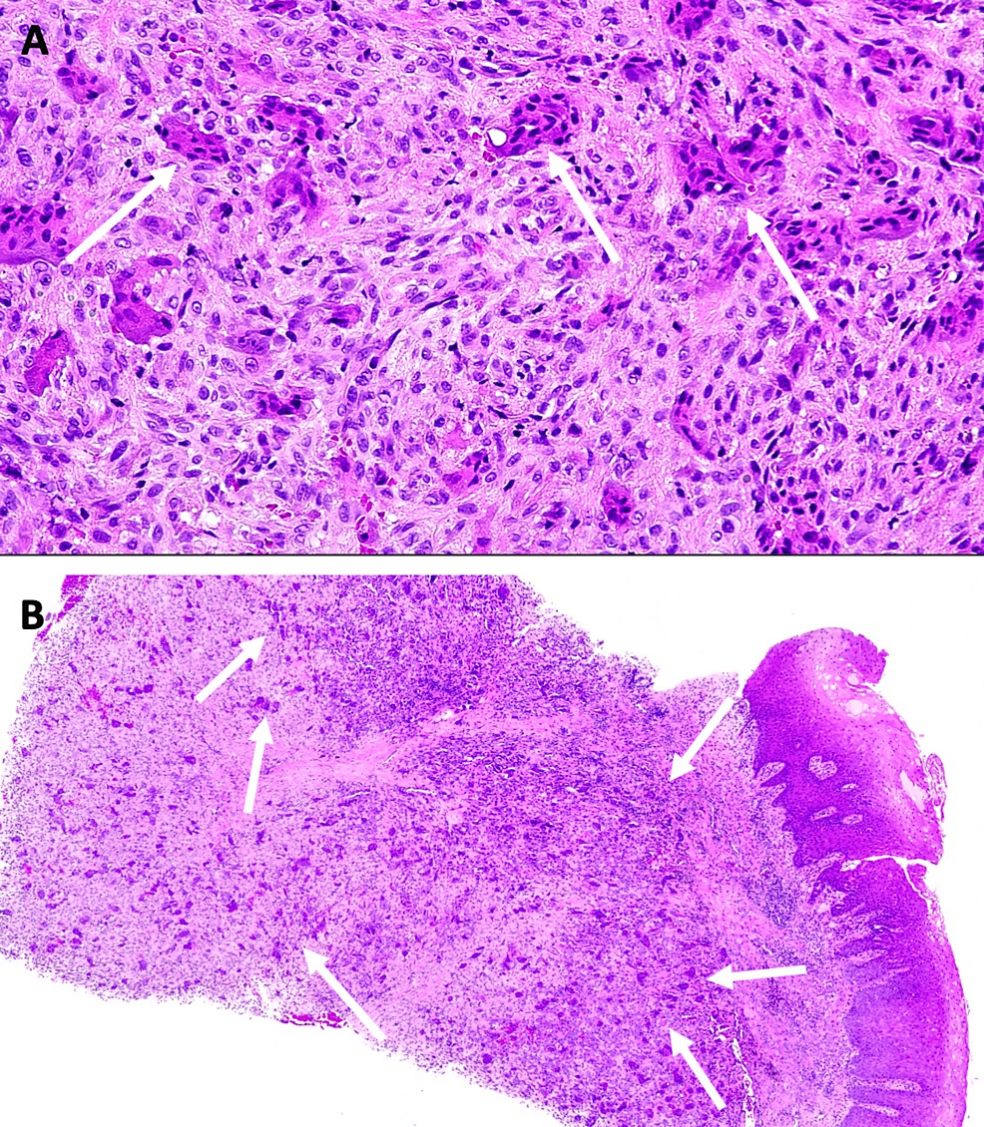
Histological sections of the lesion. (A) Several multi-nuclear cells are observed in a fibrous stroma rich in mononuclear cells (B) Multi-nuclear cells are predominantly situated in chorion.

Histologically, the appearance of these tumors is characterized by a fibrous stroma rich in mononuclear cells and a variable proportion of multinuclear cells [3]. This aspect was found on histological sections of the surgical specimen (Figure 8).

**Figure 9:**
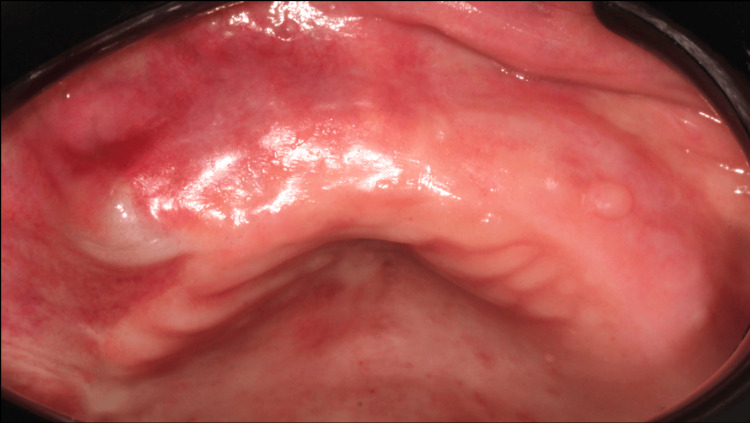
Situation four months after the surgical therapy.

After a healing time of four months, the soft tissue appears healthy and non-inflammatory. No exposure of the biomaterial or osteosynthesis screws is visible. However, primary wound closure has resulted in shortening of the vestibule, which may require additional surgery for prosthetic rehabilitation (Figure 9).

**Figure 10:**
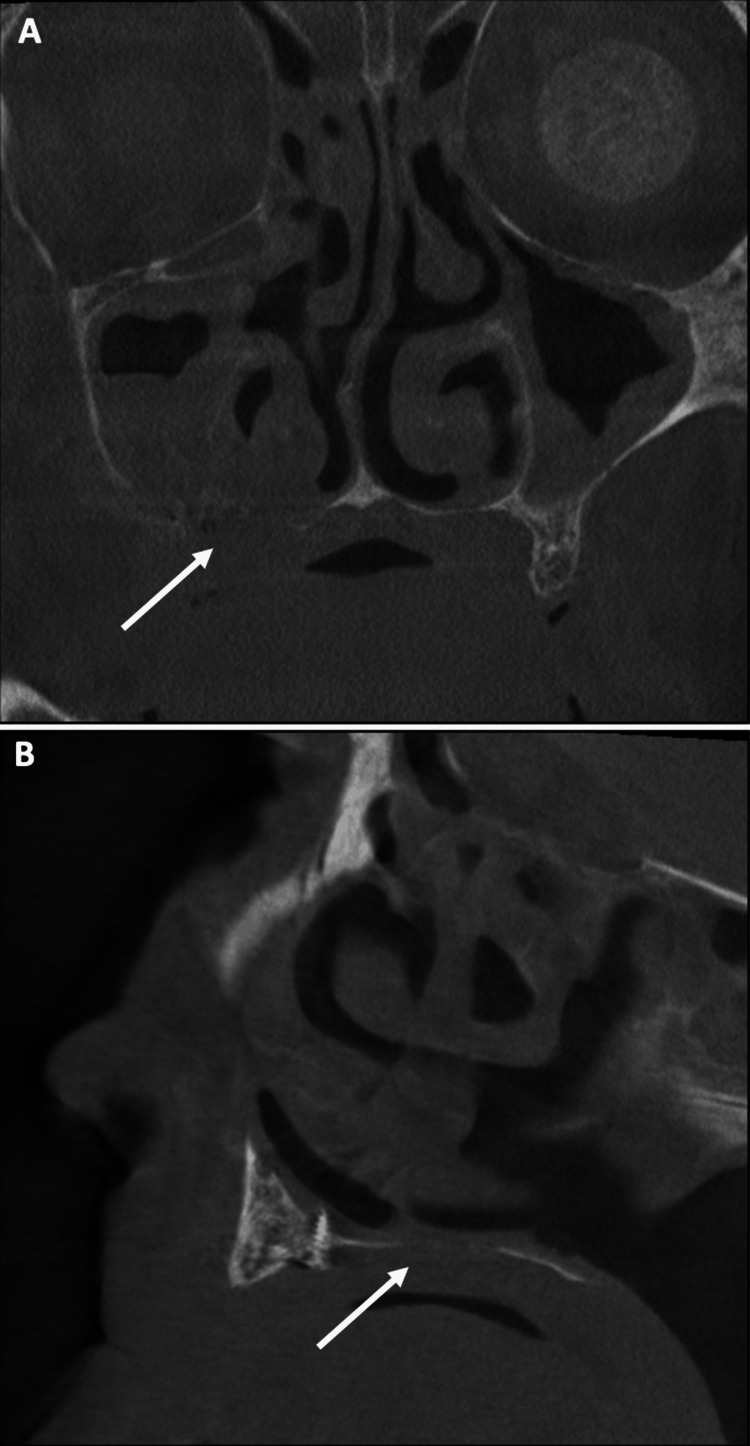
Situation at four months in (A) frontal view and (B) sagittal view of CT scan.

Despite a large and extensive bone resection, bone neoformation was achieved during the healing period. No oral or sinus symptoms were reported. This bone neoformation can be observed on CT scan (Figure 10A, 10B). An adequate healing period is essential to achieve greater bone formation and requires further radiographic controls.

## Discussion

GCTs are rare, progressive, and locally aggressive non-odontogenic osteolytic tumors associated with a high propensity for local recurrence. They represent 21% of all benign primary bone tumors, particularly in patients aged 20 to 40 years, with a slight predominance in females [7,8]. GCTs may manifest as intra- or extra-oral swelling, varying degrees of pain, tooth mobility, facial asymmetry, and a sensation of mass or pressure. These symptoms may vary depending on the location and size of the tumor. GCTs are also characterized by uni- or multilocular radiographic bone lesions of varying sizes, which may lead to cortical thinning or displacement of adjacent teeth.

Unfortunately, the etiology of GCTs remains complex and multifactorial. While genetic, environmental, and hormonal factors may contribute, the exact cause remains poorly understood. Local traumatic injuries and inflammatory conditions may also play a role in the development of these tumors [9].

The main clinical parameter for treatment success is the ability to achieve local elimination of the tumor by thorough and complete curettage [10]. While it may seem intuitive to associate the proportion of multinucleated cells observed in histopathological analysis with the aggressiveness of the lesion, Peacock et al. have reported that histopathological criteria alone are inadequate predictors of the biological behavior of GCTs [11]. To determine the nature of the lesion, it is essential to evaluate clinical, radiological and histopathological criteria. The distinction between "non-aggressive" and "aggressive" tumors is clinically important, as the latter recur more frequently after enucleation or curettage [3]. However, therapeutic modalities are the subject of considerable controversy.

Complete resection surgery to healthy margins may be performed for aggressive lesions [12], but it usually results in significant morbidity. Combined strategies involving adjuvant drugs in association with complete resection may be interesting alternatives to improve clinical outcomes, limit the necessity of extensive surgery, and prevent post-operative morbidity [1,3,13-15]. Schreuder et al. reported interesting results in bone remodeling and spontaneous bone formation in up to 90% of subjects treated with combined therapy [3].

However, random and non-predictive adverse events such as asthenia, fever, diarrhea, severe weight loss, osteonecrosis of the jaws, hypocalcemia, hypophosphatemia, or perturbation of the blood white and red cell lines could be observed, thus limiting the relevance of combined strategy due to the patient’s health condition and age [1,3].

Consequently, our treatment strategy consisted of a surgical approach based on complete resection of the lesion, followed by meticulous curettage to limit recurrence risk. However, due to the aggressiveness and progression of the lesion, resection required immediate reconstruction of the defect and intraoperative closure of buccosinusal and bucconasal communications.

The reconstruction technique needs to be decided on a case-by-case basis. The defect could be restored with bone filling using bone substitutes of various origins [10]. The concept of GBR for bone defect reconstruction, as well as for the closure of buccosinusal and bucconasal communications, led us to this therapeutic strategy. Despite the abundance of availability and lower cost of xenogeneic bone compared to allogeneic and autogenous bone, sterilization procedures considerably decrease the osteoinductive properties of the biomaterial. However, the angiogenic potential of xenogeneic substitutes promotes the formation of new blood vessels, supports the migration and differentiation of mesenchymal cells, and ensures a constant supply of oxygen essential for the proper functioning of osteoblastic and osteoclastic cells. Therefore, promising results have been observed for its use as formwork and permit us to isolate the bone component from the tissue compartment, without procurement-related patient morbidity [16].

The application of xenogeneic lamina membranes allowed us to maintain space and promoted bone healing by ensuring the stability of the blood clot. By not filling the defect, bone graft extrusion into the sinus cavity can be prevented, de novo autogenous bone formation can be achieved, and the healing process may be monitored through radiographic follow-ups. This surgical approach may complement the conventional Bichat's Ball technique, well-known for its ample vascular supply, minimal donor site morbidity, consistent surgical reliability, ease of harvesting, and low complication rate [17]. Collectively, they provide an efficient approach to closing buccosinusal and bucconasal communications and reconstructing bone defects.

Soft tissue management after bone reconstruction should also be considered. Primary wound closure resulted in scar tissue formation, which requires deepening of the vestibule prior to prosthetic rehabilitation. This treatment can improve the stability and retention of removable dentures. Deepening the vestibule provides greater support and stability at the base of the prosthesis, improving overall fit and patient comfort.

However, the treatment prognosis remains uncertain. Some results have reported a lesion recurrence rate of more than 40% after combined treatment [18]. This rate can reach 70% when the lesion is treated by curettage only [19]. Aggressive giant cell lesions of the jaws do not have a high rate of malignant transformation, but only one case has been documented in regional lymph nodes [20].

Post-surgical follow-up is crucial for evaluating mucosal healing, preventing membrane exposure (to avoid bacterial contamination), and monitoring bone formation through CT scans, with an adjusted follow-up time based on the risk of lesion recurrence.

## Conclusions

This report highlights the potential of the bone lamina technique as an attractive reconstruction strategy after the resection of GCTs. Despite the aggressiveness and extent of the lesion, this technique could improve clinical outcomes by promoting angiogenesis, providing blood supply and undifferentiated mesenchymal cells, and maintaining space for bone formation and communications closure, without procurement-related patient morbidity. However, we should not underestimate the characteristics of these lesions, which frequently require multidisciplinary treatment strategies for bone reconstruction and complex oral rehabilitation.
